# The day-7 prognostic divergence: ferritin trajectories separate survivors from non-survivors in a decade-long cohort of 9,746 hospitalised adults

**DOI:** 10.3389/fmed.2026.1952601

**Published:** 2026-10-21

**Authors:** Enrico Brunetta, Lorenzo Del Moro, Vahagn Menon, Carlo F. Selmi

**Affiliations:** 1 Rheumatology and Clinical Immunology, IRCCS Humanitas Research Hospital, Milan, Italy; 2 Department of Biomedical Sciences, Humanitas University, Milan, Italy; 3 Humanitas University, Milan, Italy

**Keywords:** biomarker trajectory, ferritin, ferritin kinetics, hospital mortality, hyperferritinaemia, macrophage activation syndrome, risk stratification, sepsis

## Abstract

**Background:**

Serial ferritin measurements have been examined in disease-specific cohorts with heterogeneous results, but the temporal pattern of ferritin in a large, clinically diverse population of hyperferritinaemic inpatients remains undefined.

**Methods:**

Retrospective single-centre cohort of adult inpatients with at least one serum ferritin ≥500 ng/mL (IRCCS Humanitas Research Hospital, October 2013–November 2024; *N* = 9,746). Thirty-day all-cause in-hospital mortality was analysed in 8,439 patients (668 deaths) by Kaplan–Meier and Cox regression adjusted for age, sex, Charlson Comorbidity Index and principal diagnosis (complete-case *n* = 6,549; 218 deaths). In a serial-sampling subgroup (*n* = 388; 387 with evaluable outcome, 45 deaths), parsimonious Cox models tested three scalar dynamic indices and mixed-effects models described log₁₀-ferritin trajectories across 0–3, 4–7 and >7 days; day-7 landmark analyses, restricted cubic splines, multiple imputation and Fine–Gray regression with live discharge as the competing event served as sensitivity analyses.

**Results:**

Thirty-day mortality rose across baseline ferritin strata (13.1%, 20.2% and 32.9%; log-rank *p* < 0.001); adjusted hazard ratios versus 500–999 ng/mL were 1.97 (95% CI 1.50–2.60) for 1,000–4,999 ng/mL and 5.46 (2.89–10.31) for ≥5,000 ng/mL. In age- and sex-adjusted longitudinal models, peak ferritin (HR 3.91 per log₁₀ unit, 95% CI 2.10–7.30; *p* < 0.001) and delta ferritin (HR 4.83, 1.60–14.57; *p* = 0.005), but not time-to-peak (*p* = 0.531), were associated with mortality. Beyond day 7, survivors' ferritin was 16.7% lower than during days 0–3 (*p* < 0.001), whereas the within-group change among patients who subsequently died was not significant; the relative difference was 15.5% (*p* = 0.020). Among 380 patients alive and hospitalised at day 7, the latest ferritin by the landmark predicted subsequent mortality (adjusted HR 2.86, 1.24–6.61; *p* = 0.014), whereas pre-landmark change, slope and any increase did not; thereafter ferritin fell by 2.30% per day in survivors but was stable in subsequent deaths (relative divergence 2.78% per day; *p* < 0.001). Spline modelling confirmed a non-linear time-by-outcome interaction (*p* = 0.036); Fine–Gray estimates were concordant.

**Conclusions:**

Ferritin level and trajectory after the first week may identify a subgroup with persistent inflammatory activity and increased subsequent mortality. Day 7 should be regarded as a candidate temporal landmark for prospective validation, not as an established clinical decision threshold. These retrospective associations do not demonstrate that ferritin-guided interventions improve outcomes.

## Background

Serum ferritin sits at the interface between iron metabolism and innate immunity. In the immunopathogenesis of inflammation, ferritin is induced by interleukin (IL)-1β, IL-6 and tumour necrosis factor (TNF)-*α* through NF-*κ*B and Nrf2 signalling, behaving as a quintessential acute-phase reactant (1–4). In a subset of life-threatening conditions - sepsis, haemophagocytic lymphohistiocytosis (HLH), macrophage activation syndrome (MAS), adult-onset Still's disease, catastrophic antiphospholipid syndrome and severe coronavirus disease 2019 (COVID-19) - extreme hyperferritinaemia is not merely a marker but an active driver of cytokine release and tissue damage, coined as the hyperferritinaemic syndromes (1, 5, 6).

In routine practice, ferritin elevation is common among hospitalised adults and encompasses chronic inflammation, hepatocellular injury and macrophage activation. This biological plurality limits its specificity but may enhance its value as a generic short-term risk marker. Admission hyperferritinaemia has been associated with adverse outcomes in transplantation, intensive-care, sepsis, oncology and COVID-19 cohorts (7–14). Serial measurements have also been studied, but with heterogeneous findings: early ICU ferritin changes added little to admission values in a large COVID-19 cohort, whereas early declines were associated with survival in secondary haemophagocytic syndrome, day-7 ferritin outperformed admission ferritin in a multicentre polytrauma cohort, and longitudinal ferritin was not associated with mortality in a joint model of maintenance haemodialysis patients (15–18). Whether a reproducible temporal divergence exists across a large, heterogeneous population of hyperferritinaemic inpatients remains unclear.

We therefore hypothesised that longitudinal ferritin trajectories would differ between survivors and non-survivors and that this difference could be localised to a clinically interpretable temporal window. We further examined whether the first ferritin ≥500 ng/mL and conventional scalar summaries of kinetics (time-to-peak, peak and delta ferritin) were associated with short-term all-cause in-hospital mortality. To analyse heterogeneous free-text records accumulated over more than a decade, we used a Python natural-language-processing (NLP) pipeline to calculate the Charlson Comorbidity Index (CCI) and assign principal diagnoses, in line with current methodological standards for the high-throughput generation of real-world evidence (19–22).

## Methods

### Study design and setting

We conducted a single-centre, retrospective observational cohort study at IRCCS Humanitas Research Hospital, a tertiary academic hospital in Rozzano (Milan), Italy. The study screened all admissions between 1 October 2013 and 30 November 2024 (≈11 years) and was reported in accordance with the Strengthening the Reporting of Observational Studies in Epidemiology (STROBE) statement for cohort studies (23). The protocol was approved by the local Institutional Review Board and the requirement for individual informed consent was waived for the secondary use of de-identified electronic health record (EHR) data, consistent with Italian regulations on retrospective observational research.

### Population

Eligible patients were adults (aged ≥18 years) admitted to any medical or surgical ward or intensive-care unit during the study period who had at least one serum ferritin measurement ≥500 ng/mL during the reference hospitalisation. The threshold of 500 ng/mL was chosen because it consistently identifies clinically significant hyperferritinaemia across reference laboratories and is in line with the lower limit used in the literature on hyperferritinaemic syndromes (1, 5, 6). For patients with multiple admissions, only the first eligible admission was considered. Fifty-one patients carrying four residual labels of the diagnosis-revision step (rheumatological, *n* = 13; neurological, *n* = 8; nephrological, *n* = 22; unclassified, *n* = 8) were dropped when the analytical dataset was built, because these labels were too small to be modelled as stand-alone categories in the six-category scheme used for the longitudinal analyses. Since the exclusion was applied to the dataset as a whole, it also affected the cross-sectional cohort, leaving 9,746 unique patients, even though the three named labels have a natural counterpart among the 11 cross-sectional categories (immuno-rheumatological, neuro-psychiatric and renal/urinary). The eight unclassified records carried free-text principal diagnoses that the pipeline could not map to any analytical category. The 43 patients with the three named labels represent 0.44% of the otherwise eligible cohort (43/9,789); their exclusion is acknowledged as a limitation. The specific conditions that contribute most to the highest ferritin values are described in the Discussion.

The cohort was operationally divided into (a) patients in whom ferritin was measured only once during hospitalisation (*n* = 6,383); and (b) patients with multiple measurements during the reference hospitalisation (*n* = 3,363, indexed to the first eligible value). To enable the longitudinal modelling of biologically meaningful trajectories, a further subgroup (*n* = 388) was extracted from group (b) after applying pre-defined eligibility criteria, which are described in detail below.

### Exposure and outcome definitions

The exposure of interest was the first ferritin value ≥500 ng/mL measured during the index hospitalisation. Ferritin was modelled both as a three-level ordinal variable (500–999, 1,000–4,999, ≥5,000 ng/mL) and, on a continuous scale, after log₁₀-transformation to address right-skew. The primary outcome was 30-day all-cause in-hospital mortality, ascertained from administrative discharge records. The time origin was the date of the first qualifying ferritin determination, with administrative censoring at hospital discharge or 30 days, whichever came first. Patients discharged alive on the same day as the first qualifying ferritin determination, or with missing exit date, were excluded from the survival analysis (*n* = 1,307), yielding 8,439 patients at risk with 668 deaths in 110,489 person-days of observation.

### Covariates

Covariates were demographic (age and sex), clinical (CCI and principal discharge diagnosis) and pharmacological. The CCI was derived from problem-list and admission-note free text by a Python NLP pipeline based on regular-expression matching of International Classification of Diseases (ICD)-10/ICD-9-CM-aligned condition phrases extended with Italian-language synonyms. Principal diagnoses were aggregated into 11 categories in the primary cohort (infectious, cardiovascular, neuro-psychiatric, respiratory, immuno-rheumatological, haematological, gastrointestinal/hepatic, renal/urinary, musculoskeletal, endocrine/metabolic, oncological) and into six categories in the longitudinal subgroup (respiratory, infectious, HLH/MAS, haematological, COVID-19 and oncological) to limit sparse cells. COVID-19 was therefore modelled as a separate category in the longitudinal analyses. Exposure to drug classes known to influence ferritin (systemic corticosteroids, antibiotics, non-corticosteroid immunosuppressants) was represented by time-fixed binary indicators of in-hospital administration.

### Validation of the natural-language-processing pipeline

To assess the accuracy of the automated extraction, 200 index admissions were randomly selected from the primary cohort (*n* = 9,746) and reviewed manually by a clinician (L.D.M.) blinded to the pipeline output. For each record, the reviewer derived the Charlson Comorbidity Index and assigned the principal-diagnosis category from the clinical record, which served as the reference standard. Agreement was defined at record level as concordance of both the total CCI score and the principal-diagnosis category with manual review and is reported as a proportion with a 95% Wilson confidence interval. Condition-level sensitivity and specificity, separate agreement statistics for the CCI and for the diagnosis, and inter-rater reliability were not assessed; this is acknowledged as a limitation.

### Eligibility criteria for the longitudinal subgroup

The longitudinal subgroup was defined by (i) ≥6 serum ferritin measurements during the reference hospitalisation; (ii) a mean interval between consecutive measurements of 1–5 days; and (iii) a ferritin-monitoring span, from the first to the last qualifying measurement, of ≤30 days. The third criterion concerns the monitoring span rather than the length of stay. These criteria operationally selected patients undergoing repeated monitoring, and did not guarantee two observations in every time bin. Of 3,363 patients with multiple measurements, 388 met all criteria. For each patient, three dynamic indices were calculated on the log₁₀ scale: (a) time-to-peak - days from the first value ≥500 ng/mL to the day of the maximum log₁₀ ferritin value (the first occurrence in the case of a tie); (b) ferritin peak - the maximum log₁₀ ferritin value observed during hospitalisation; and (c) ferritin delta - the peak log₁₀ ferritin value minus the first log₁₀ ferritin value. Patient-level descriptors of sampling intensity (number of determinations, monitoring span and mean inter-sample interval) are summarised as medians (IQR). Patients who met and who did not meet the eligibility criteria were compared to quantify selection into the longitudinal subgroup.

### Statistical analysis

#### Descriptive statistics

Continuous variables are presented as mean ± standard deviation (SD) or median (interquartile range, IQR), depending on distribution; categorical variables as counts (%). The skewed ferritin distribution (mean 1,420.5 ± 2,533.9 ng/mL; median 857 ng/mL, IQR 627–1,416) motivated routine log₁₀-transformation for inferential modelling (mean log₁₀ 3.01, SD 0.28). Stratum-level comparisons used Pearson χ^2^ tests for categorical variables and the Shapiro–Wilk test to confirm non-normality before log-transformation.

#### Cross-sectional analysis

Survival was estimated using the Kaplan–Meier method, and the strata were compared using the log-rank test. Adjusted hazard ratios (HRs) with 95% confidence intervals (CI) were obtained from multivariable Cox proportional hazards regression, with the following covariates: age (continuous variable, with HRs reported in 10-year increments for greater clinical interpretability), sex, CCI (continuous variable) and primary diagnosis categorised into 11 categories. The proportional hazards assumption was assessed using scaled Schoenfeld residuals, both overall and for each covariate.

#### Longitudinal subgroup analyses

For the longitudinal survival analyses, follow-up began at the first qualifying ferritin measurement and ended at in-hospital death, live discharge or 30 days, whichever occurred first; in the originally submitted analysis, follow-up had erroneously been terminated at the last ferritin measurement, and all longitudinal survival results have been re-estimated with the corrected definition. Because the originally submitted fully adjusted model was severely over-fitted, the primary Cox models examined one dynamic index at a time with adjustment for age and sex; 1,000 non-parametric bootstrap replications assessed coefficient stability. A COVID-19 versus non-COVID-19 term was added in sensitivity analyses; the six-category diagnosis could not be used in the Cox models because some categories contained no deaths. The original complete-case model containing all three indices, CCI, six-category diagnosis and three drug classes was retained only as a transparency analysis and was not used for inference. Proportional-hazards assumptions were assessed using scaled Schoenfeld residuals, and a coefficient varying with log-time was fitted where the assumption was violated.

Ferritin trajectories were modelled with patient-specific random-intercept linear mixed-effects models; same-day determinations were averaged so that each patient contributed at most one observation per day. Time was discretised *a priori* into 0–3, 4–7 and >7 days, reflecting the conventional clinical week-1 transition from peak innate-immune activation to resolution or progression of hyperinflammation (24, 25); the partition was fixed before analysis and was not derived from the data. The primary categorical model included mortality-by-time interactions with adjustment for age, sex and the six-category diagnosis. The interaction was re-estimated in a minimally adjusted model (age and sex), after adding drug-class exposures, and - for transparency only - in models including the CCI, which retain few deaths because of outcome-dependent CCI missingness; a further sensitivity analysis used any in-hospital death as the outcome. Post-week-1 within-group changes and between-group contrasts were back-transformed from log₁₀ coefficients to percentage differences.

#### Missing data

Missingness was summarised at patient level and compared by 30-day outcome. Complete-case sample attrition was reported sequentially. Because CCI and drug-exposure missingness was strongly outcome-dependent, missing completely at random was not assumed. Multiple imputation by chained equations used 100 imputations: predictive mean matching for the CCI and augmented logistic regression for the drug indicators, with age, sex, six-category diagnosis, baseline log₁₀ ferritin, the three dynamic indices, the outcome indicator and the Nelson–Aalen cumulative hazard included in the imputation model. The analysis models were Cox models including one dynamic index, age, sex and the imputed CCI, combined by Rubin's rules. These estimates were treated as sensitivity analyses because the fractions of missing information were high.

#### Landmark, continuous-time and competing-risk analyses

A day-7 landmark analysis included only patients alive and still hospitalised immediately after day 7; follow-up was re-started at the landmark, so that no immortal person-time before day 7 contributed to the analysis. Cox models adjusted for age and sex evaluated the latest ferritin available by day 7, the change from baseline, the mean pre-landmark slope and any increase from baseline. A mixed model adjusted for age, sex and COVID-19 status then compared post-landmark slopes between survivors and subsequent deaths. Restricted cubic splines with four knots (placed at days 0, 4, 10 and 21) modelled continuous time in a mixed model adjusted for age, sex and COVID-19 status; time-by-outcome interactions tested whether trajectories differed, and time-specific contrasts were back-transformed to adjusted geometric-mean ferritin ratios (subsequent deaths versus survivors). Landmark analyses were repeated at days 5 and 10. Finally, Fine–Gray regression treated live discharge as a competing event for in-hospital death in the longitudinal cohort.

#### Software and significance

All analyses were performed in Stata/IC 15.1 (StataCorp LLC, College Station, TX). Two-sided *p*-values <0.05 were considered statistically significant. The Python NLP pipeline was developed in Python 3.x.

## Results

### Cohort characteristics

Between October 2013 and November 2024, 9,746 inpatients had at least one serum ferritin ≥500 ng/mL after exclusion of four small principal-diagnosis categories (51 patients in total). Men accounted for 66.9% (6,520/9,746) of the cohort, the mean age was 67.25 ± 14.92 years (median 69.4, IQR 58.4–78.4), and the median CCI was 2 (IQR 1–4). The median first ferritin value was 857 ng/mL (IQR 627–1,416), with a maximum of 163,343 ng/mL (log₁₀ ≈ 5.21); the distribution was strongly right-skewed (Shapiro–Wilk *p* < 0.001 for raw values), justifying log-transformation for inferential modelling (Supplementary Figure S1). Baseline characteristics by ferritin stratum are summarised in Table 1. Ferritin strata could be assigned to 9,745 of the 9,746 eligible patients (5,777, 3,686 and 282, respectively); one eligible record lacked a valid first ferritin value on the log₁₀ scale and is therefore absent from the stratum-specific columns of Table 1 and from Supplementary Figure S1. Across ascending ferritin strata, mean age declined from 68.3 years (500–999 ng/mL) to 62.3 years (≥5,000 ng/mL) and the male proportion declined from 67.4% to 50.0%. The highest stratum therefore had equal male and female representation; these descriptive data do not establish that women were more likely to have extreme hyperferritinaemia.

**Table 1:** Baseline characteristics of the study cohort, overall and by ferritin stratum at first determination (*n* = 9,746).

Variable	Overall (*N* = 9,746)	500–999 ng/mL (*n* = 5,777)	1,000–4,999 ng/mL (*n* = 3,686)	≥5,000 ng/mL (*n* = 282)
Age, years, mean ± SD	67.25 ± 14.92	68.30 ± 14.87	65.96 ± 14.76	62.29 ± 15.60
Age, years, median (IQR)	69.4 (58.4–78.4)	70.6 (59.3–79.7)	67.7 (57.3–77.0)	65.3 (53.4–73.3)
Male sex, *n* (%)	6,520 (66.9)	3,891 (67.4)	2,488 (67.5)	141 (50.0)
Ferritin, ng/mL, mean ± SD	1,420.5 ± 2,533.9	688.7 ± 138.3	1,883.8 ± 895.3	10,354.9 ± 10,854.1
Ferritin, ng/mL, median (IQR)	857 (627–1,416)	659 (569–793)	1,527 (1,222–2,280)	6,818 (5,541–9,886)
log₁₀(ferritin), mean ± SD	3.014 ± 0.283	2.830 ± 0.085	3.235 ± 0.179	3.916 ± 0.245
Charlson comorbidity index*, median (iqr)	2 (1–4)	2 (1–4)	2 (1–4)	2 (2–4)
Charlson comorbidity index*, mean ± SD	2.94 ± 2.70	2.89 ± 2.72	3.02 ± 2.66	2.93 ± 2.80
Overall in-hospital mortality, *n* (%)	1,778 (18.2)	872 (15.1)	815 (22.1)	91 (32.3)
30-day in-hospital mortality (KM), %	–	13.1	20.2	32.9

Stratum columns sum to 9,745 because one eligible record lacked a valid first ferritin value on the log₁₀ scale (see Results and Supplementary Figure S1). Mean age and the male proportion declined across ascending strata; the ≥5,000-ng/mL stratum was younger and had equal male and female representation. Overall in-hospital mortality differed across strata (Pearson χ^2^ = 112.1 on 2 df, *p* < 0.001).

* CCI computed by the Python natural-language-processing pipeline; CCI was available in 7,747/9,746 patients (79.5%) overall, 4,815/5,777 (83.4%) in the 500–999 stratum, 2,761/3,686 (74.9%) in the 1,000–4,999 stratum and 171/282 (60.6%) in the ≥5,000 stratum. Thirty-day cumulative in-hospital mortality is the Kaplan–Meier estimate from the survival cohort (*n* = 8,439). IQR, interquartile range; SD, standard deviation; KM, Kaplan–Meier.

### Cross-sectional analysis: ferritin at first determination and 30-day mortality

Of the 9,746 eligible patients, 8,439 provided valid data on the duration of participation in the study for the survival analysis, following the exclusion of same-day discharges and medical records with a missing discharge date (*n* = 1,307 excluded); 668 deaths occurred within 30 days of the baseline ferritin measurement. Kaplan–Meier curves showed a clear separation between the three ferritin levels (log-rank χ^2^ = 73.46 on 2 df, *p* < 0.001; Figure 1). The cumulative 30-day in-hospital mortality, estimated by the Kaplan–Meier function at *t* = 30, increased monotonically: 13.1% (95% CI 11.6–14.8) for 500–999 ng/mL, 20.2% (95% CI 18.0–22.6) for 1,000–4,999 ng/mL and 32.9% (95% CI 24.4–43.4) for ≥5,000 ng/mL. In the fully adjusted multivariable Cox model (*n* = 6,549, 218 events; restricted to medical records with complete information on the CCI and primary diagnosis), the adjusted HRs (95% CI) relative to the reference value of 500–999 ng/mL were 1.97 (1.50–2.60; *p* < 0.001) for the 1,000–4,999 ng/mL range and 5.46 (2.89–10.31; *p* < 0.001) for values ≥5,000 ng/mL (Table 2). Age was independently associated with increased mortality (HR per 10 years 1.66, 95% CI 1.47–1.88; *p* < 0.001) while sex was not (HR for male sex 1.10, 95% CI 0.82–1.46; *p* = 0.528). The CCI displayed an inverse association with mortality (HR 0.85 per point, 95% CI 0.80–0.91; *p* < 0.001). The global Schoenfeld test was borderline (χ^2^ = 23.85 on 15 df, *p* = 0.068); two diagnosis-level covariates (respiratory and haematological) showed individual residual time-dependence (*p* = 0.001 and *p* = 0.028 respectively), without invalidating the global model.

**Figure 1:**
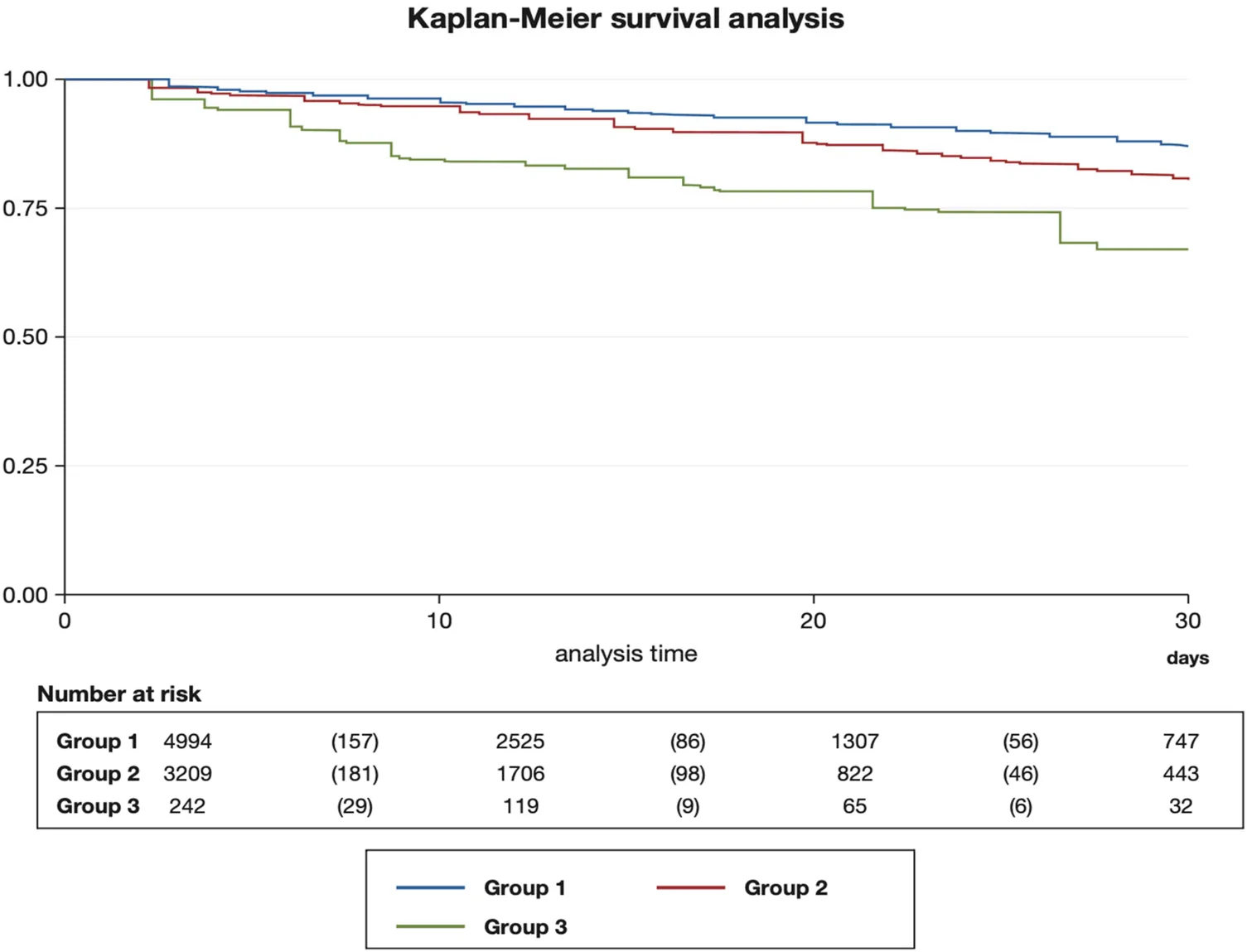
Kaplan–Meier 30-day in-hospital survival curves, stratified by serum ferritin at first determination (*n* = 8,439; 668 events). Survival probability over the 30 days following the first ferritin ≥500 ng/mL, stratified into three ordinal strata: 500–999 ng/mL (Group 1; *n* = 4,991; 299 deaths; 30-day survival 0.869, 95% CI 0.852–0.885), 1,000–4,999 ng/mL (Group 2; *n* = 3,206; 325 deaths; 30-day survival 0.798, 95% CI 0.774–0.820) and ≥5,000 ng/mL (Group 3; *n* = 242; 44 deaths; 30-day survival 0.671, 95% CI 0.566–0.756). The endpoint was all-cause in-hospital death. A monotonic gradient is evident, with significantly lower survival in higher strata (log-rank χ^2^ = 73.46 on 2 df, *p* < 0.001). The number-at-risk table is shown beneath the curves.

**Table 2:** Multivariable cox proportional-hazards model for 30-day in-hospital mortality in the primary survival cohort (*n* = 6,549; 218 events).

Covariate	Adjusted HR	95% CI	*p*-value
Ferritin 500–999 ng/mL	1.00 (ref.)	–	–
Ferritin 1,000–4,999 ng/mL	1.97	1.50–2.60	<0.001
Ferritin ≥5,000 ng/mL	5.46	2.89–10.31	<0.001
Age (per 10 years)	1.66	1.47–1.88	<0.001
Sex (male vs. female)	1.10	0.82–1.46	0.528
Charlson Comorbidity Index (per point)	0.85	0.80–0.91	<0.001
Principal diagnosis (11-category)	–	–	Included as fixed effects*

Hazard ratios from a multivariable Cox proportional-hazards model adjusted simultaneously for ferritin stratum, age, sex, CCI and 11-category principal diagnosis. Likelihood-ratio χ^2^ = 222.03 on 15 df (*p* < 0.001). The global Schoenfeld test was borderline (χ^2^ = 23.85 on 15 df; *p* = 0.068), with residual time-dependence for the respiratory and haematological diagnosis categories.

* Diagnosis-category HRs versus the infectious-disease reference (selected): respiratory HR 1.83 (95% CI 1.28–2.63), haematological HR 0.29 (0.16–0.56), musculoskeletal HR 0.08 (0.02–0.32); other categories non-significant. The inverse CCI coefficient should not be interpreted causally; possible explanations include selection/index-event bias, residual confounding by acute illness severity, coding limitations, NLP misclassification and differential missingness (see Discussion). HR, hazard ratio; CI, confidence interval; ref., reference.

### Longitudinal subgroup: dynamic indices and survival

The longitudinal subgroup comprised 388 patients; 387 had an evaluable 30-day outcome, with 45 in-hospital deaths over 7,803 person-days of observation (mean follow-up 20.2 days). Patients underwent a median of 7 ferritin determinations (IQR 6–9) over a median monitoring span of 16 days (IQR 12–21), with a median mean inter-sample interval of 2.4 days (IQR 1.8–3.0). In univariable Cox regression, time-to-peak was not associated with mortality (HR 0.986 per day, 95% CI 0.947–1.026; *p* = 0.479), peak ferritin was associated with mortality (HR 2.52 per log₁₀ unit, 1.31–4.84; *p* = 0.005), and delta ferritin was not statistically significant (HR 2.55, 0.90–7.18; *p* = 0.077). In the primary age- and sex-adjusted models, time-to-peak remained null (HR 0.988, 0.950–1.027; *p* = 0.531), whereas peak ferritin (HR 3.91, 2.10–7.30; *p* < 0.001) and delta ferritin (HR 4.83, 1.60–14.57; *p* = 0.005) were associated with higher mortality (Table 3). Bootstrap 95% confidence intervals were 0.95–1.02 for time-to-peak, 2.06–7.43 for peak and 1.43–16.24 for delta ferritin. Results were similar after adding COVID-19 status and after multiple imputation. The proportional-hazards assumption was satisfied for time-to-peak and peak ferritin but not for delta ferritin (Schoenfeld *p* = 0.028); a model with a delta coefficient varying with log-time did not show a significant change over follow-up (*p* = 0.106), and the delta estimate should be interpreted as an average effect over the 30-day window.

**Table 3:** Cox proportional-hazards models for 30-day in-hospital mortality in the outcome-evaluable longitudinal subgroup (*n* = 387; 45 deaths; 7,803 person-days).

Dynamic index	Univariable HR (95% CI; p)	Primary adjusted HR* (95% CI; p)	COVID-19 sensitivity**	MI sensitivity***
Time-to-peak, per day	0.986 (0.947–1.026; 0.479)	0.988 (0.950–1.027; 0.531)	0.986 (0.948–1.025; 0.477)	0.991 (0.952–1.032; 0.665)
Peak ferritin, per log₁₀ unit	2.521 (1.314–4.838; 0.005)	3.912 (2.098–7.298; <0.001)	4.363 (2.112–9.016; <0.001)	3.934 (2.078–7.446; <0.001)
Delta ferritin, per log₁₀ unit	2.549 (0.905–7.180; 0.077)	4.826 (1.599–14.572; 0.005)	4.597 (1.483–14.246; 0.008)	4.882 (1.626–14.657; 0.005)

* Follow-up runs from the first qualifying ferritin measurement to in-hospital death, live discharge or day 30. *Primary models include one dynamic index at a time and adjust for age and sex (45 deaths; 3 coefficients; 15 events per coefficient). Bootstrap 95% confidence intervals (1,000 replications) were 0.952–1.024 for time-to-peak, 2.061–7.428 for peak and 1.434–16.241 for delta ferritin.

** Sensitivity model additionally adjusts for COVID-19 versus non-COVID-19 diagnosis (*n* = 383; 45 deaths).

*** Multiple imputation by chained equations with 100 imputations (*n* = 383; 45 deaths), analysis model adjusted for age, sex and the imputed Charlson Comorbidity Index; largest fraction of missing information 0.76. The proportional-hazards assumption was not met for delta ferritin (Schoenfeld *p* = 0.028), so its HR is an average over follow-up. The originally submitted fully adjusted complete-case model (288 patients, 7 deaths, 14 coefficients) is not reported because it cannot support inference. HR, hazard ratio; CI, confidence interval; MI, multiple imputation.

The originally submitted fully adjusted complete-case model was not used for inference. Correcting the survival time and requiring complete CCI, diagnosis and drug data left 288 patients and only 7 deaths. With 14 fitted coefficients, the events-per-parameter ratio was approximately 0.5. CCI missingness was the principal source of attrition: only 8 of 45 deaths had an observed CCI.

### Post-week-1 divergence in ferritin trajectories

The primary categorical mixed model included 2,964 patient-day observations from 383 patients (Figure 2, Table 4). Relative to days 0–3, survivor ferritin was unchanged during days 4–7 (*β* = 0.014, 95% CI −0.005 to 0.033; *p* = 0.161) and lower beyond day 7 (*β* = −0.079, −0.096 to −0.063; *p* < 0.001), corresponding to a 16.7% reduction. The death-by->7-day interaction was positive (*β* = 0.063, 0.014–0.112; *p* = 0.012), a 15.5% relative difference versus survivors (*p* = 0.020). However, the combined within-group change among patients who subsequently died was small and non-significant (*β* = −0.017, 95% CI −0.063 to 0.029; *p* = 0.474; −3.8%). Thus, the revised model supports failed resolution, or relative persistence, of ferritin among subsequent deaths rather than a statistically demonstrated re-escalation. The interaction was similar in the minimally adjusted model (387 patients, 45 deaths; *β* = 0.058, *p* = 0.020) and when any in-hospital death was used as the outcome (52 deaths; *β* = 0.060, *p* = 0.009); after further adjustment for drug-class exposures (329 patients, 35 deaths) it was attenuated and no longer significant (*β* = 0.052, 95% CI −0.006 to 0.109; *p* = 0.080), and models including the CCI retained only seven or eight deaths and were not used for inference.

**Figure 2:**
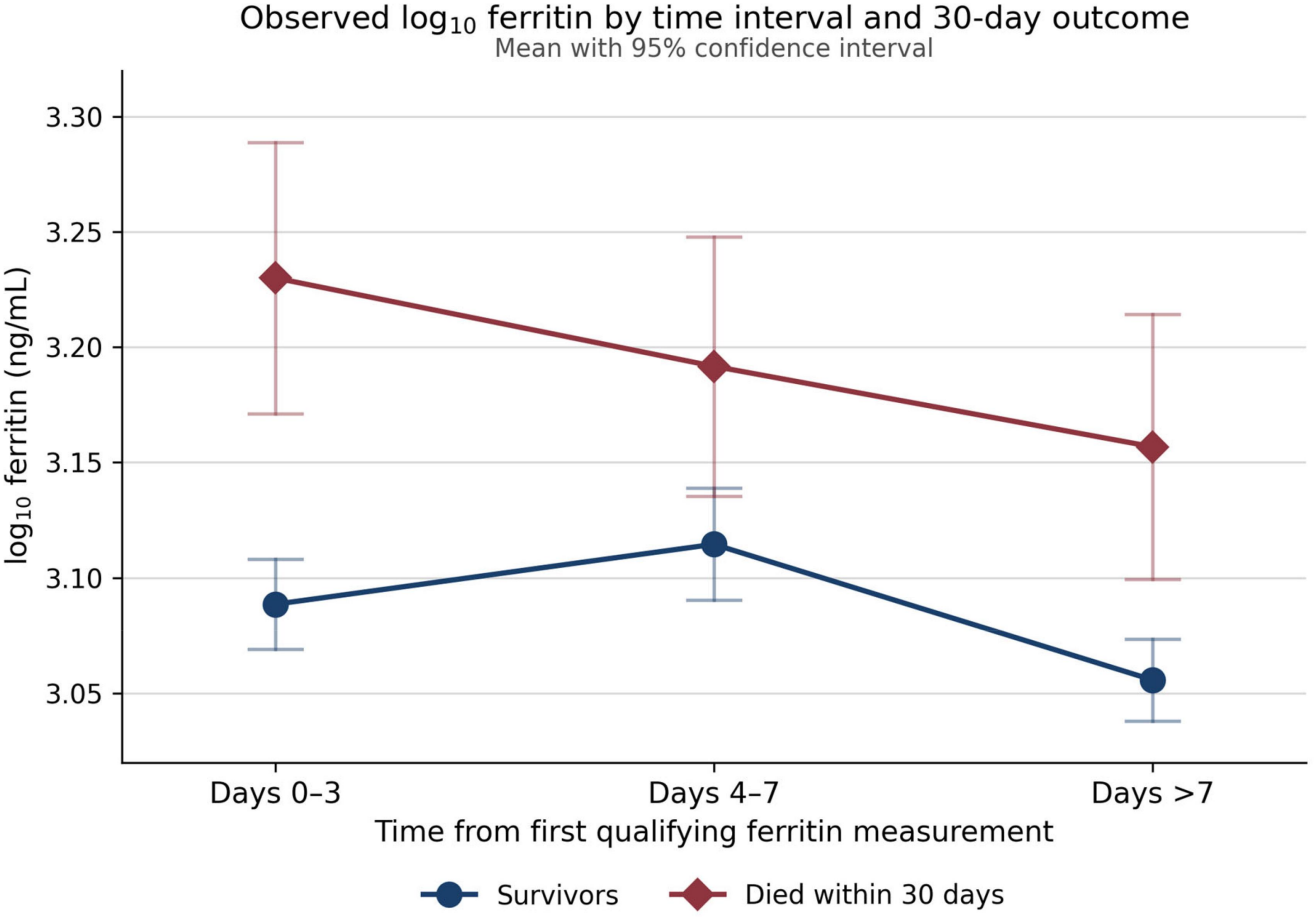
Observed log₁₀ ferritin by time interval and 30-day outcome in the longitudinal subgroup. Mean log₁₀ ferritin with 95% confidence intervals across the 0–3, 4–7 and >7-day intervals among survivors (circles) and patients who died within 30 days (diamonds), based on all 3,000 determinations from the 387 patients with an evaluable outcome. Survivors showed a decline beyond day 7, whereas ferritin among patients who subsequently died remained relatively persistent. These are descriptive means; the corresponding model-based estimates, adjusted for age, sex and diagnosis, are reported in Table 4. Values beyond day 7 are conditional on remaining alive, hospitalised and under ferritin monitoring.

**Table 4:** Primary categorical mixed-effects model of log₁₀ ferritin (2,964 patient-day observations from 383 patients; 45 deaths).

Term	*β* (log₁₀)	95% CI	*p*-value
Time bin 0–3 days	0 (reference)	–	–
Time bin 4–7 days (survivors)	+0.014	−0.005 to +0.033	0.161
Time bin >7 days (survivors)	−0.079	−0.096 to −0.063	<0.001
Death (intercept shift, days 0–3)	+0.086	+0.012 to +0.160	0.023
Death × time bin 4–7 days	−0.020	−0.074 to +0.034	0.471
Death × time bin >7 days	+0.063	+0.014 to +0.112	0.012
Age, per year	−0.0008	−0.0025 to +0.0008	0.328
Sex, male	+0.026	−0.026 to +0.078	0.331
Principal diagnosis (six categories)	–	–	Included as fixed effects

Patient-specific random-intercept model adjusted for age, sex and six-category principal diagnosis; same-day determinations were averaged so that each patient contributes at most one observation per day. Coefficients are on the log₁₀ scale. Beyond day 7, the survivor coefficient corresponds to a 16.7% reduction from the days 0–3 reference, and the mortality interaction to a 15.5% relative difference (*p* = 0.020). The net within-group change among patients who subsequently died was −0.017 log₁₀ (95% CI −0.063 to 0.029; *p* = 0.474), corresponding to −3.8%, and does not support a significant re-escalation. Random-intercept SD 0.199; residual SD 0.178.

### Sensitivity and robustness analyses

#### Selection and missingness

Of the 3,363 patients with multiple measurements, 388 met the longitudinal criteria and 2,975 did not. Reasons for exclusion were not mutually exclusive: fewer than six measurements (*n* = 2,747), mean interval outside 1–5 days (*n* = 1,226), monitoring span >30 days (*n* = 430) and no non-negative interval (*n* = 3). Included and excluded patients had similar age (median 67.3 vs. 66.8 years; *p* = 0.975), but included patients were more often male (76.6% vs. 68.1%; *p* = 0.001), had slightly higher baseline log₁₀ ferritin (*p* = 0.045), a lower observed CCI (median 1 vs. 2; *p* < 0.001) and a different length-of-stay distribution (median 22 vs. 20 days; *p* = 0.014). Thirty-day mortality did not differ significantly when missing outcomes were retained as a category (11.6% vs. 9.1%; *p* = 0.234). Diagnosis and drug-exposure comparisons were not interpretable outside the selected cohort because these variables were largely unavailable there.

At patient level, the CCI was missing in 57/388 (14.7%), diagnosis in 5/388 (1.3%) and each drug indicator in 57/388 (14.7%); one outcome was unavailable. CCI missingness was strongly associated with death: 37/45 deaths (82.2%) versus 19/342 survivors (5.6%) lacked a CCI (*p* < 0.001), and patients without a CCI were older (mean 73.3 vs. 64.1 years). Sequential complete-case samples were 387 patients/45 deaths for the core variables, 383/45 after adding diagnosis, 329/35 after adding drug exposure, 327/8 after adding the CCI, and 288/7 for the fully complete dataset. In 100-imputation sensitivity analyses (*n* = 383, 45 deaths), estimates remained null for time-to-peak (HR 0.99, 95% CI 0.95–1.03; *p* = 0.665) and positive for peak (HR 3.93, 2.08–7.45; *p* < 0.001) and delta ferritin (HR 4.88, 1.63–14.66; *p* = 0.005). The largest fraction of missing information was 0.76, so these imputed estimates are supportive rather than definitive.

#### Day-7 landmark analysis

Seven patients had been discharged or had died by day 7, including two deaths, leaving 380 patients and 43 subsequent deaths. The latest log₁₀ ferritin available by day 7 predicted subsequent mortality after adjustment for age and sex (HR 2.86, 95% CI 1.24–6.61; *p* = 0.014; unadjusted HR 1.93, 0.84–4.45; *p* = 0.122). Change from baseline (HR 0.63; *p* = 0.517), mean pre-landmark slope (HR 1.30; *p* = 0.941) and any increase from baseline (HR 0.74; *p* = 0.337) were not associated with subsequent mortality. In the post-landmark mixed model (1,543 observations from 368 patients), ferritin decreased by 2.30% per day among survivors (*p* < 0.001) but remained statistically stable among subsequent deaths (+0.42% per day; *p* = 0.480); the relative between-group difference was +2.78% per day (95% CI 1.50–4.05; *p* < 0.001). These results condition explicitly on survival and continued hospitalisation to day 7.

#### Continuous-time and alternative-landmark analyses

Restricted cubic splines showed a significant overall time-by-mortality interaction (χ^2^ = 20.84 on 3 df; *p* = 0.0001), improved fit over a linear-time model (likelihood-ratio χ^2^ = 75.10 on 4 df; *p* < 0.001), and evidence that the mortality-related divergence was itself non-linear (likelihood-ratio χ^2^ = 6.67 on 2 df; *p* = 0.036). Adjusted ferritin ratios for subsequent deaths versus survivors were above one on days 0–2 (day 0 ratio 1.30, 95% CI 1.07–1.58), crossed the null during days 3–6, and became significant again from day 7 onward (day 7 ratio 1.215, 95% CI 1.019–1.448; day 30 ratio 1.749, 1.223–2.503) (Figure 3). At the day-5 landmark (387 patients, 45 deaths), the latest ferritin level was associated with subsequent mortality (HR 3.31, 95% CI 1.29–8.48; *p* = 0.013); the corresponding estimate at day 10 (346 patients, 38 deaths) was HR 3.01 (1.36–6.66; *p* = 0.006). Post-landmark mixed models showed the same qualitative pattern, with survivor declines and relative between-group slope differences of 2.88% per day at day 5 and 3.16% per day at day 10 (both *p* < 0.001), while the within-death slopes were not statistically significant. These analyses support day 7 as a candidate landmark but do not establish it as an optimal threshold.

**Figure 3:**
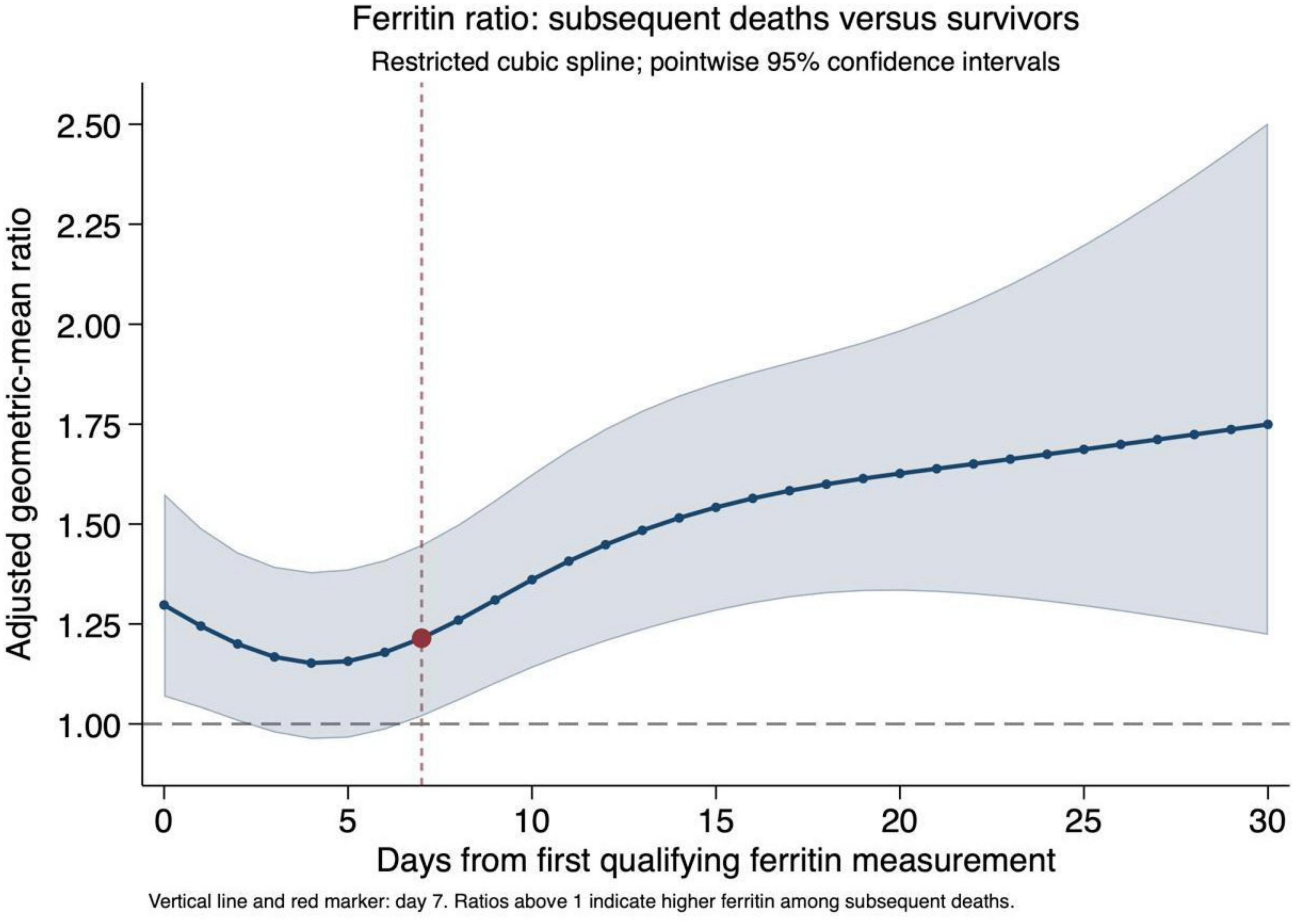
Adjusted ferritin ratio between subsequent deaths and survivors over time (restricted cubic spline). Adjusted geometric-mean ratio of ferritin in patients who died within 30 days versus survivors, estimated day by day from the mixed-effects model with restricted cubic splines of time (knots at days 0, 4, 10 and 21), adjusted for age, sex and COVID-19 status (383 patients; 2,964 patient-day observations), with pointwise 95% confidence intervals. The dashed vertical line and the red marker indicate day 7. Ratios above one indicate higher ferritin among subsequent deaths; the ratio was above one on days 0–2, crossed the null during days 3–6 and was significantly above one from day 7 onward. Pointwise intervals are descriptive and do not establish an optimised clinical cut-off.

#### Competing risk of live discharge

Among the 387 patients there were 45 deaths, 276 live discharges and 66 administrative censorings at 30 days. Fine–Gray results were concordant with the primary Cox analyses: subdistribution HRs were 1.01 for time-to-peak (95% CI 0.97–1.05; *p* = 0.649), 3.52 for peak ferritin (1.98–6.23; *p* < 0.001) and 4.60 for delta ferritin (1.55–13.69; *p* = 0.006). The delta effect attenuated over time (time-interaction subdistribution HR 0.155; *p* = 0.027).

### Validation of the natural-language-processing pipeline

In the 200 randomly selected index admissions from the primary cohort, the pipeline output agreed with blinded manual review on both the total CCI score and the principal-diagnosis category in 186 records (93.0%, 95% CI 88.6–95.8%). In the remaining 14 records (7.0%) at least one of the two elements differed from manual review; the nature of these discrepancies was not characterised further.

## Discussion

In this retrospective cohort of 9,746 hospitalised adults, the first ferritin value ≥500 ng/mL was associated with a dose-dependent gradient of 30-day in-hospital mortality, independent of age, sex, comorbidity and principal diagnosis. In the corrected longitudinal analysis, peak and delta ferritin - but not time-to-peak - were associated with mortality in parsimonious models. More importantly, the categorical, landmark and spline analyses consistently showed that patients who survived experienced ferritin resolution after the first week, whereas ferritin remained relatively persistent among patients who subsequently died. The evidence supports a post-week-1 divergence, but not the original claim of a statistically demonstrated re-escalation among non-survivors.

The timing is biologically plausible. The end of the first week marks the transition from peak innate-immune activation to either resolution or progression into a hyperinflammatory syndrome; in sepsis, severe COVID-19, post-transplant complications, HLH and MAS, days 5–10 are when cytokine release either subsides or evolves into refractory inflammation and organ failure (5, 6, 24–26). A ferritin trajectory that fails to decline beyond this window may therefore be a parsimonious marker of failed resolution, although our data cannot distinguish this from the effect of unmeasured illness severity or treatment.

These results help place prior disease-specific evidence in context. In 2,265 patients hospitalised with COVID-19, Shakaroun et al. found prognostic value for admission ferritin but little added value from serial ICU measurements during days 1–4 (15). Koymen et al. reported that early ferritin decreases within the first treatment week were associated with survival in secondary haemophagocytic syndrome (16). In a multicentre polytrauma cohort, Chen et al. found that day-7 ferritin discriminated mortality better than day-1 ferritin and that non-survivors sustained hyperferritinaemia while survivors showed a steeper decline (17). Outside the acute setting, Li et al. modelled longitudinal log-ferritin jointly with survival in 254 maintenance haemodialysis patients and found no significant association with mortality, underlining that the prognostic meaning of ferritin kinetics depends on the clinical context (18). Our findings also align with earlier evidence in allogeneic transplantation, sepsis and severe COVID-19 (7–14, 27, 28). Differences in disease mix, sampling intensity, timing and treatment plausibly explain some of the heterogeneity. Our contribution is therefore not the first study of ferritin kinetics, but the characterisation of a non-linear temporal pattern in a broad, unselected hyperferritinaemic inpatient cohort spanning ward and intensive-care patients.

The day-7 landmark analysis changes the clinical interpretation. Among patients alive and hospitalised at day 7, the contemporaneous ferritin level was associated with subsequent mortality, whereas the preceding delta, slope and binary increase were not. After the landmark, ferritin declined among survivors but was statistically stable among subsequent deaths. Thus, the clinically relevant signal may be the failure of ferritin to resolve rather than an increase *per se*. The spline and day-5/day-10 sensitivity analyses indicate that this pattern is not an artefact of one arbitrary partition, although they do not prove that day 7 is an optimal threshold.

The revised scalar-index findings differ from the original report because the survival time was corrected to run from the first qualifying ferritin measurement to death, live discharge or day 30, rather than to the last ferritin measurement. Peak and delta ferritin were associated with mortality after age and sex adjustment and in multiple-imputation and competing-risk analyses. The delta estimate requires caution because the proportional-hazards assumption was not met and the association attenuated over time in the competing-risk model. The severely over-fitted original complete-case model, with seven events for 14 coefficients, cannot support inference; the attenuation of the trajectory interaction after adjustment for drug exposures in the smaller drug-complete subsample likewise counsels caution.

The demographic gradients deserve cautious interpretation. Men predominated overall, but the male proportion fell to 50% in the ≥5,000-ng/mL stratum; this indicates equal sex representation at the highest values, not female predominance. Patients in that stratum were also younger. These patterns may reflect the diagnostic composition of the selected cohort, local ferritin-testing practices and enrichment for acute hyperinflammatory or haematological conditions rather than intrinsic age- or sex-specific susceptibility. The available data do not permit causal attribution.

The composition of the extreme-ferritin range illustrates why the broad diagnostic categories should be read with care. Among the 112 patients with a ferritin ≥10,000 ng/mL, the most frequent admission diagnoses were acute myeloid leukaemia (27%), other lymphomas (16%), solid tumours (14%), Hodgkin lymphoma (9%), other leukaemias (8%), COVID-19 (7%), multiple myeloma (7%), sepsis (6%), adult-onset Still's disease (4%) and acute kidney injury (2%), whereas the most frequent discharge diagnoses were infectious complications after haematopoietic stem-cell transplantation (36%), other infectious complications (30%), HLH (16%), MAS (6%) and graft-versus-host disease (4%). In the range where ferritin is most strongly associated with mortality, the haematological category is therefore dominated by patients in whom transfusion-related iron overload, hepatic involvement and post-transplant infection or graft-versus-host disease coexist with hyperinflammation. The haematological, gastrointestinal/hepatic and renal/urinary categories thus capture non-inflammatory as well as inflammatory determinants of ferritin, and their coefficients in the adjusted models should be interpreted as category-level adjustments rather than as estimates for homogeneous conditions.

The inverse CCI association in the cross-sectional model is unlikely to represent a protective effect. Collider or index-event bias from conditioning on ferritin ≥500 ng/mL is one possible explanation (29, 30): because the cohort is conditioned on ferritin elevation, high-comorbidity patients often cross the threshold through chronic disease-related elevation, whereas low-comorbidity patients with ferritin ≥500 ng/mL are enriched for acute aetiologies carrying very high short-term mortality. This mechanism is, however, not directly demonstrated. Residual confounding by acute illness severity, selection effects, incomplete diagnostic coding and misclassification by the NLP pipeline (record-level agreement with manual review 93%, but condition-level accuracy not assessed) are plausible alternatives, and the completeness of the free text from which the index was derived varied markedly across strata (79.5% overall but 60.6% in the ≥5,000 ng/mL stratum). The strong outcome-dependent CCI missingness in the longitudinal subgroup further cautions against causal interpretation of CCI coefficients, which should be regarded as uninterpretable in causal terms.

Several limitations remain. Post-day-7 measurements are conditional on survival, continued hospitalisation and clinical decisions to re-measure ferritin; the landmark analysis removes immortal person-time before day 7 but cannot eliminate informative observation thereafter. A joint longitudinal–survival model was considered but not fitted, because sparse events and outcome-dependent covariate missingness would make such a model unstable in this sample. The serial-sampling criteria selected a small, intensively monitored subgroup that differed from other patients with repeated measurements in sex, baseline ferritin, comorbidity and length of stay. Live discharge is informative; Fine–Gray analyses reduce, but do not remove, this concern. Missingness was strongly outcome-dependent, especially for the CCI, and high fractions of missing information limit the certainty of multiple imputation. The NLP-derived covariates showed 93% record-level agreement with blinded manual review in a random sample of 200 admissions, but condition-level performance and inter-rater reliability were not assessed, so residual, and possibly differential, misclassification may still affect adjusted estimates. Forty-three patients with rheumatological, neurological or nephrological labels (0.44% of the otherwise eligible cohort) were excluded at data preparation rather than merged into the corresponding cross-sectional categories; given their number, this cannot have materially affected the ferritin hazard ratios estimated from 8,439 patients and 668 deaths, but the dataset would have been more complete had they been retained. The day-7 partition, although pre-specified on biological grounds, is one of several defensible choices and is not claimed to be optimal. Finally, this was a single tertiary centre in northern Italy, in a predominantly European population, and included only patients with ferritin ≥500 ng/mL; the results may not generalise to community hospitals, non-European populations with different genetic and epidemiological determinants of iron metabolism and inflammation, different testing practices, or patients with lower ferritin values. COVID-19 was modelled separately in the longitudinal analyses, but the pandemic altered admission and testing practices during part of the study period. Set against these limitations, the strengths of the study are the large, unselected, decade-long sample, the inclusion of both ward and intensive-care patients, and the joint cross-sectional and longitudinal analysis with comorbidity adjustment at scale.

In conclusion, ferritin measured around and after the first week may identify patients with persistent inflammatory activity and increased subsequent mortality risk. Day 7 should be treated as a candidate temporal landmark for external and prospective validation rather than as an established clinical decision point. The study demonstrates association only and does not show that source-control reassessment, HLH/MAS screening, immunomodulation or escalation of care triggered by ferritin trajectories improves outcomes. Future multicentre studies should validate the trajectory in geographically diverse populations, integrate concurrent biomarkers and test whether ferritin-guided reassessment changes patient outcomes.

## Data Availability

The raw data supporting the conclusions of this article will be made available by the authors, without undue reservation.
