# Supplementary material for: The day-7 prognostic divergence: ferritin trajectories separate survivors from non-survivors in a decade-long cohort of 9,746 hospitalised adults
Source: Front Med (Lausanne). 2026 Oct 21;13:1952601. doi: 10.3389/fmed.2026.1952601 (PMC13731380; doi:10.3389/fmed.2026.1952601)
Supplement: Supplementary file 1 [file Supplementaryfile1.docx]

**Supplementary Figure S1**

**Supplementary Figure S1**. Distribution of log₁₀(serum ferritin) at first determination in the study cohort (N=9,745). Histogram of log₁₀-transformed values for the first qualifying ferritin determination during admission. Log-transformation was applied to address strong right-skew of the raw distribution (median 857 ng/mL, IQR 627–1,416 ng/mL; Shapiro–Wilk W = 0.239, p<0.001 for raw values). On the log₁₀ scale, the mean is 3.014 (SD 0.283) and the median 2.93 (IQR 2.80–3.15). The inclusion threshold (500 ng/mL) corresponds to log₁₀ ≈ 2.70; the right-hand tail reflects rare extreme values (maximum 163,343 ng/mL, log₁₀ ≈ 5.21).
